# Sepsis caused by emphysematous pyelonephritis: A case report

**DOI:** 10.3389/fmed.2022.1038455

**Published:** 2023-01-06

**Authors:** Zheng Yang, Zhihui Li

**Affiliations:** Department of Intensive Care Unit, Hangzhou Red Cross Hospital, Hangzhou, Zhejiang, China

**Keywords:** case report, sepsis, infection, diabetes, emphysematous pyelonephritis (EPN)

## Abstract

**Purpose:**

Emphysematous pyelonephritis (EPN) is a rare, life-threatening necrotizing renal parenchymal infection. It is most commonly reported in patients with poor glycemic control.

**Patient concerns:**

We report the case of a 64-year-old woman who presented to the emergency room with fever and weakness over the last few days.

**Diagnosis:**

After a series of tests in the diagnostic workup, the patient was diagnosed with emphysematous pyelonephritis and sepsis.

**Intervention and outcome:**

She received conservative treatment with meropenem and symptomatic treatment, and the symptoms improved significantly.

**Lessons:**

EPN can be reliably diagnosed using non-contrast abdominal CT imaging. The infection is most commonly caused by the *Escherichia coli* species, and a good curative effect can be achieved with early diagnosis and appropriate and timely treatment.

## 1. Introduction

Emphysematous pyelonephritis (EPN) is an acute, severe necrotizing infection resulting in gas in the renal parenchyma, collecting system, or perinephric tissue. EPN is uncommon due to its rare characteristics. Literature on the most effective treatment approach for EPN is scarce. Recently, after reviewing the literature, the researchers emphasized that the symptoms of this illness demonstrate unique pathology and point to microbiological and epidemiological causes (1). The condition was prolonged in this case and was carefully managed with good clinical effects.

We report a case of EPN in a 64-year-old female patient with type 2 diabetes mellitus, which was confirmed using computed tomography (CT) and laboratory evaluations.

## 2. Case report

**Figure 1:**
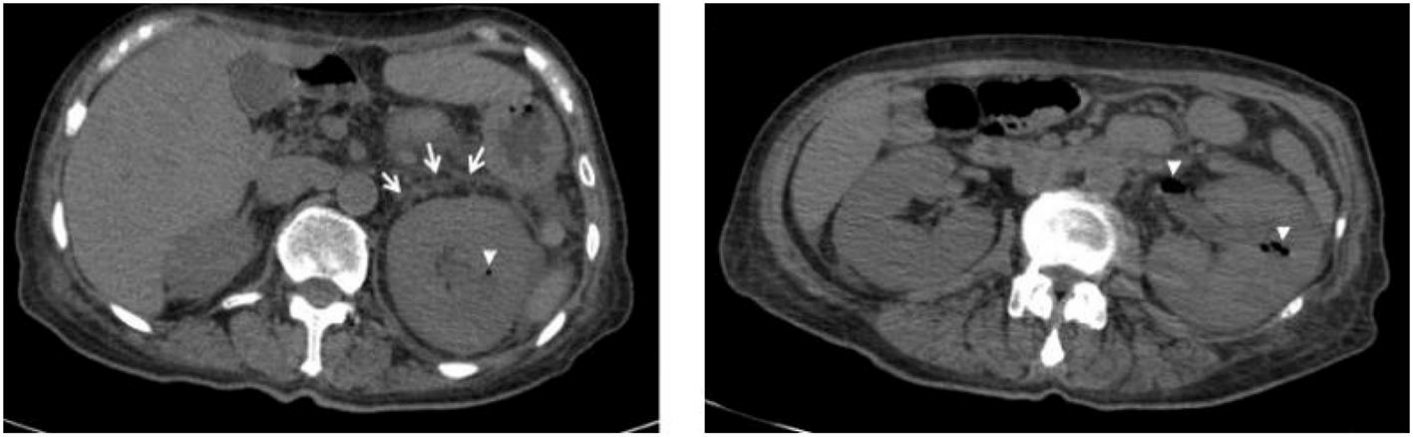
CT scan revealed gas collection in the parenchyma and renal pelvis (arrowheads) and perirenal fascia thickening (arrows) of the left kidney.

A 64-year-old woman presented to the emergency room with fever and weakness over the last few days. The patient was feeling agitated during the physical examination. She also complained of left loin pain. Her medical history included poorly controlled type II diabetes mellitus and chronic renal failure. Laboratory results demonstrated that her white cell count was 27,700 cells per cubic millimeter (the normal range: 3,500 to 9,500). The C-reactive protein level was 32.873 mg per deciliter, the blood urea nitrogen level was 20.70 mmol per liter, and the creatinine level was 312.7 μmol per liter. Urinalysis showed an elevated leukocyte count and bacteriuria. A CT scan in horizontal plane sectioning showed that the left kidney was significantly enlarged with thickened perirenal fascia, which was related to the presence of gas in the renal parenchyma and the kidney pelvis (Figure 1). She was transferred to the intensive care unit and treated with meropenem and broad-spectrum empirical antibiotics.

**Figure 2:**
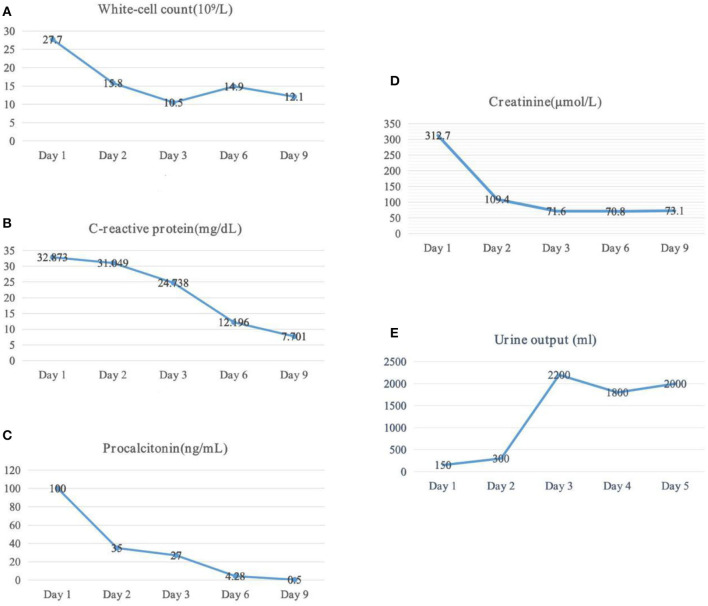
Laboratory tests and urine output dynamic change. **(A)** Changes of white-cell, **(B)** changes of c-reactive protein, **(C)** changes of procalcitonin, **(D)** changes of serum creatinine, and **(E)** changes of urine output.

On admission to the ICU (day 0), she was drowsy. Her vital signs were as follows: body temperature, 37.5°C; pulse rate, 102 beats/min; respiratory rate, 28 breaths/min; blood pressure, 122/54 mmHg (noradrenaline 0.5 microg/kg per min); and pulse oxygen saturation, 98% with high flow oxygen therapy. Diminished breath sounds in the right lower lung were heard on auscultation. There was no audible cardiac murmur. Her abdomen was soft but she failed to cooperate with the abdominal tenderness examination, along with mild pitting edema of both lower limbs. The patient was diagnosed with emphysematous pyelonephritis and septic shock, possibly caused by a gas-producing uropathogenic infection. Unfortunately, according to the RIFLE criteria (2), the patient developed acute renal failure and was treated with continuous renal replacement therapy. However, after active treatment, the clinical effect was remarkable. Her clinical symptoms and serum inflammatory indicators (Figures 2A–C) improved gradually, and she stopped using norepinephrine (day 3). Her renal function (day 3) also recovered (Figures 2D, E), and she was successfully weaned with continuous renal replacement therapy. On the third day, both blood and urine cultures reported extended-spectrum beta-lactamase (ESBL)-producing *E. coli*. The patient's condition continued to improve, and she no longer needed to be treated in the ICU on the 6^th^ day. The patient's abdominal CT scan (day 12) revealed that the gas collection in the pelvis and calyces had disappeared. Finally, the patient was discharged.

## 3. Discussion

After admission, the patient's EPN was diagnosed according to clinical manifestations, laboratory examinations, and abdominal CT scan results. Septic shock and acute renal failure occurred. We empirically gave anti-infection treatment, and the patient received appropriate supportive treatment while controlling the blood sugar at the appropriate level.

Currently, an abdominal CT scan is the preferred radiographic method for diagnosing EPN and staging its severity, which is related to its treatment plan (3). Huang et al. classified EPN into four classes: Class 1 indicates gas confined to the collecting system; Class 2 indicates gas confined to the renal parenchyma without extension to the extrarenal space; Class 3A indicates extension of gas or abscess to the perinephric space; Class 3B pertains to extension of the gas or abscess to the pararenal space; and Class 4 refers to bilateral EPN or a solitary kidney with EPN (4). Our patient was judged to have a Class 3A EPN.

A recent meta-analysis found that the mortality rate among patients with EPN disease was 13%, with a significantly decreasing trend in mortality rates from 1985 to 2020 (5). EPN was first defined by Schultz and Klorfein (6), and they opposed open surgical drainage of multiple cortical abscesses. The doctors' constant supervision revealed compelling results. Compared with conservative treatment, patients with diabetes mellitus who underwent surgical drainage or nephrectomy had lower mortality (7). Since then, open surgery has played a central role in the management of EPN, and the results from statistics from Shah's study showed that open surgery was associated with high mortality (8). A report published 10 years later by Chen et al. recommended different procedures. Antibiotic therapy combined with CT-guided percutaneous drainage for the treatment of emphysematous pyelonephritis is an appropriate alternative to antibiotic therapy with surgical intervention (9). With the advent of modern imaging, endourological procedures, and broad-spectrum antibiotics, the majority of such patients can be treated with minimal morbidity and mortality, even with the salvaging of the renal units (10, 11).

The disease usually occurs in female patients with diabetes with poor glycemic control, with or without urinary tract obstruction (12). *E. coli* is the most common pathogen (13). Almost 70% of patients extract *E. coli* from urine or pus cultures. Our case combines in-hospital multidisciplinary consultation methods, including urologists, critical care physicians, and microbiologists, to manage patients with EPN and develop strategies that may produce the best survival results. The first step includes full fluid resuscitation, broad-spectrum parenteral antibiotics against gram-negative bacteria, correction of electrolyte and acid-base disorders, and strict blood glucose control. We dynamically evaluated the function of each organ, such as the heart, the lung, and the kidney and provided emergency support treatment when necessary, including percutaneous drainage, positive muscle strength, mechanical ventilation, continuous hemodialysis, and so on. In cases of intractable shock or persistent bacteremia, nephrectomy should be considered.

## 4. Conclusion

Emphysematous pyelonephritis is an uncommon, acute, and severe necrotizing infection. Due to the rarity and variability of the clinical symptoms of EPN, diagnosing EPN proves to be a challenge. EPN can be reliably diagnosed by non-contrast CT abdominal imaging. The case study we report prove that surgical treatment of EPN is inappropriate in patients with serious complications, and the prognosis of patients may be improved by an early and regular course of antibacterial treatment.

## Data availability statement

The original contributions presented in the study are included in the article/supplementary material, further inquiries can be directed to the corresponding author.

## Ethics statement

The studies involving human participants were reviewed and approved by Medical Ethics Committee of Hangzhou Red Cross Hospital. The patients/participants provided their written informed consent to participate in this study.

## Author contributions

ZY: data curation, funding acquisition, and writing—original draft. ZY and ZL: investigation. ZL: resources and writing—review and editing. Both authors contributed to the article and approved the submitted version.
